# Ethanol Extracts of Fresh *Davallia formosana* (WL1101) Inhibit Osteoclast Differentiation by Suppressing RANKL-Induced Nuclear Factor-**κ**B Activation

**DOI:** 10.1155/2013/647189

**Published:** 2013-09-28

**Authors:** Tzu-Hung Lin, Rong-Sen Yang, Kuan-Chin Wang, Dai-Hua Lu, Houng-Chi Liou, Yun Ma, Shao-Han Chang, Wen-Mei Fu

**Affiliations:** ^1^Department of Pharmacology, College of Medicine, National Taiwan University, No. 1 Section 1 Jen-Ai Road, Taipei 10051, Taiwan; ^2^Department of Orthopedics, National Taiwan University Hospital, No. 7 Chung-Shan S Road, Taipei 10002, Taiwan; ^3^Won-Lin Pharmaceutical Company, No. 104, Feng-Ping 1st Road, Kaohsiung City 83145, Taiwan

## Abstract

The rhizome of *Davallia formosana* is commonly used to treat bone disease including bone fracture, arthritis, and osteoporosis in Chinese herbal medicine. Here, we report the effects of WL1101, the ethanol extracts of fresh rhizomes of *Davallia formosana* on ovariectomy-induced osteoporosis. In addition, excess activated bone-resorbing osteoclasts play crucial roles in inflammation-induced bone loss diseases, including rheumatoid arthritis and osteoporosis. In this study, we examined the effects of WL1101 on receptor activator of nuclear factor-**κ**B ligand (RANKL)-induced osteoclastogenesis. Treatment with WL1101 significantly inhibited RANKL-stimulated osteoclastogenesis. Two isolated active compounds, ((−)-epicatechin) or WL14 (4-hydroxy-3-aminobenzoic acid) could also inhibit RANKL-induced osteoclastogenesis. WL1101 suppressed the RANKL-induced nuclear factor-**κ**B (NF-**κ**B) activation and nuclear translocation, which is the key process during osteoclastogenesis, by inhibiting the activation of I**κ**B kinase (IKK) and I**κ**B**α**. In animal model, oral administration of WL1101 (50 or 200 mg/kg/day) effectively decreased the excess bone resorption and significantly antagonized the trabecular bone loss in ovariectomized rats. Our results demonstrate that the ethanol extracts of fresh rhizomes of *Davallia formosana* inhibit osteoclast differentiation via the inhibition of NF-**κ**B activation and effectively ameliorate ovariectomy-induced osteoporosis. WL1101 may thus have therapeutic potential for the treatment of diseases associated with excessive osteoclastic activity.

## 1. Introduction

Bone is an important organ which can provide mechanical support to soft tissues such as brain and spinal cord. Bone participates in maintaining blood calcium, phosphate, and hematopoiesis as well. Both osteoblast-mediated synthesis and osteoclast-mediated resorption contribute to the dynamic remodeling process in bone tissue [1, 2]. Osteoblasts are derived from mesenchymal stem cells and are responsible for bone matrix formation including bone mineralization [1]. On the other hand, multinucleated osteoclasts which are differentiated from hematopoietic cells are responsible for bone resorption [2]. Both cell types are regulated by circulating hormones, mechanical stress, and inflammatory mediators. Once the amount of osteoclast-mediated bone resorption is more than that of bone formation, then osteoporosis ensures.

Differentiation of osteoclasts is regulated by the interactions among multiple molecules within the bone microenvironment provided by osteoblasts and stromal cells [2]. Two hematopoietic factors mainly regulate osteoclastogenesis, the TNF-related cytokine RANKL (receptor of activation of nuclear factor kappa B ligand), which can activate osteoclasts and is proposed to regulate osteoclast differentiation from precursor cells through NF-*κ*B signaling pathway, and M-CSF (macrophage colony stimulating factor) [2, 3]. In addition, osteoclasts can respond to inflammatory mediators. Excess activation of osteoclasts is involved in many diseases such as osteoporosis and arthritis. Furthermore, osteoclast-mediated bone resorption plays a pivotal role in bone metastasis of cancer including breast, prostate, colorectal, and lung cancer [4]. 

Inhibition of the excess activation of osteoclast is the therapeutic strategy for many bone diseases including osteoporosis and rheumatoid arthritis. The rhizome of *Drynaria fortunei* is one of the Chinese herbal medicines for the treatment of bone diseases. It has been reported that the extract of rhizome of *Drynaria fortunei* can inhibit the survival, differentiation, and resorption of osteoclasts [5–7]. The rhizome of *Davallia formosana* was also used to treat bone diseases as *Drynaria fortunei*. It is also reported that the extracts of rhizome of *Davallia formosana* can inhibit the differentiation and activation of osteoclasts [8]. 

In this study, the ethanol extract (WL1101) of fresh rhizome of *Davallia formosana* was used. In order to show the possible effects of WL1101 on bone cells. We also purified the active pure compound from WL1101, and it was found that WL13 and WL14 can significantly inhibit RANKL-induced osteoclastogenesis. WL1101 was orally administered to ovariectomized (OVX) rats and markedly ameliorated OVX-induced bone resorption and bone loss.

## 2. Materials and Methods

### 2.1. Animals

 All protocols complied with institutional guidelines and were approved by Animal Care Committees of Medical College, National Taiwan University. Three-month-old female Wistar rats (230–270 g) were purchased from BioLASCO Taiwan Co., Ltd. (I-Lan, Taiwan).

### 2.2. Materials

Recombinant mouse RANKL (462-TEC: amino acids 158–317 expressed in *Escherichia coli*) and mouse M-CSF were purchased from R&D system (Minneapolis, MN, USA). Fetal bovine serum was from Biological Industries (Kibbutz Beit Haemek, Israel). A murine monocytic cell line, RAW264.7, was obtained from American Type Culture Collection (ATCC; Manassas, VA). Rabbit polyclonal antibody for IKK*α*/*β*, NF-*κ*B p50, or NF-*κ*B p65, mouse monoclonal antibody for C23, and goat anti-mouse or anti-rabbit secondary antibody conjugated with horseradish peroxidase were purchased from Santa Cruz Biotechnology (Santa Cruz, CA, USA). Mouse monoclonal antibody for phospho-I*κ*B*α* and rabbit monoclonal antibody for phospho-IKK*α*/*β* were purchased from Cell Signaling Technology (Danvers, MA, USA). Mouse monoclonal antibody for actin was purchased from Merck-Millipore (Bedford, MA, USA). The *α*-MEM and antibiotics were obtained from Gibco Invitrogen (Carlsbad, CA, USA). 3-(4,5-dimethyl thiazol-2-yl)-2,5-diphenyl tetrazolium bromide (MTT), *β*-glycerophosphate, L-ascorbic acid, cetylpyridinium chloride, collagenase, p-nitrophenol phosphate disodium, and tartrate-resistant acid phosphatase (TRAP) staining kit were purchased from Sigma-Aldrich (St. Louis, MO, USA). Plants of *Davallia formosana* were provided by Won-Lin Pharmaceutical Company. The fresh rhizomes of *Davallia formosana* were extracted using ethanol to obtain WL1101. Active compounds, WL13 and WL14, were separated by using HPLC (Hitachi High-Tech, Tokyo, Japan; Column C18 4.6 ∗ 250 mm).

### 2.3. Primary Culture of Osteoclasts Derived from Bone Marrow Macrophages

Primary osteoclasts were derived from bone marrow macrophages (BMMs) in femurs of 8–12-week-old (300–400 g) male Wistar rats [9]. Bone marrow cells were harvested by flushing the bone marrow cavity with *α*-MEM. Cells were cultured in *α*-MEM supplemented with 10% heat-inactivated fetal bovine serum (FBS), 100 U/mL penicillin, and 100 *μ*g/mL streptomycin (pH adjusted to 7.6). After 24 h's incubation, the non-adherent cells (primary bone marrow macrophages) were collected. Cells were plated at a density of 10^6^ per well (0.5 mL) with recombinant soluble RANKL (5 ng/mL) and M-CSF (20 ng/mL) at 37°C in 5% CO_2_ in humidified air. After 5 days' culture, the cells were washed with PBS twice and then fixed with 4% paraformaldehyde for 2 min. Cells were stained with a TRAP staining kit at 37°C for 1 h in the dark chamber. Cells were then washed with distilled water and air dried for photography and counting. TRAP-positive cells with more than 3 nuclei were defined as osteoclasts. 

### 2.4. Primary Osteoblast Cultures

Primary osteoblastic cells were obtained from the calvaria of 1-day-old Wistar rats [10]. In brief, the calvaria of fetal rats was dissected with aseptic technique, and the soft tissue was removed under dissecting microscope. The calvaria was then divided into small pieces and treated with collagenase solution (1 mg/mL) for 20–30 min at 37°C. The next two 20 min sequential collagenase digestions were then pooled and filtered through 70 *μ*m nylon filters (Falcon, BD Biosciences, San Jose, CA, USA). The cells were grown on plastic cell culture dishes in 95% air-5% CO_2_ with *α*-MEM, which was supplemented with 10% heat-inactivated FBS, 100 U/mL penicillin, and 100 *μ*g/mL streptomycin (pH adjusted to 7.6). The cell medium was changed every 3 days. The characteristics of osteoblasts were confirmed by morphology and the expression of alkaline phosphatase (ALP).

### 2.5. Measurement of Cell Viability by MTT Assay

 MTT was used as an indicator of cell viability as determined by its mitochondria-dependent reduction to formazan. Osteoblasts were plated onto 24-well culture plates at a density of 2 × 10^4^ cells/well in 10% FBS/*α*-MEM. Cells were grown for 48 h and then treated with various concentrations of WL1101 for 2 days. After washing the cells by PBS, 200 *μ*L *α*-MEM containing 0.5 mg/mL MTT was added to each well. Cells were incubated for 30 min at 37°C, the supernatant was then removed, and the formed blue crystals in viable cells were solubilized with dimethylsulfoxide (DMSO), which was transferred to 96-well plate, and the absorbance of each well was measured at 550 nm by using a microplate reader (Bio-Tek, Winooski, VT, USA).

### 2.6. Measurement of Calcium Deposition in Osteoblasts

Osteoblasts were plated onto 24-well culture plates at a density of 2 × 10^4^ cells/well in 10% FBS/*α*-MEM supplemented with 50 *μ*g/mL L-ascorbic acid and 10 mM *β*-glycerophosphate [10]. The culture medium was changed every three days, and test substances were newly replaced. After 2 weeks, the cells were washed twice with TBS, fixed in ice-cold 75% (vol/vol) ethanol for 30 min, and then air dried. Calcium deposition was determined using quantitative alizarin red S staining. In brief, the ethanol-fixed cells and matrix were stained for 1 h with 40 mM alizarin red-S (pH 4.2) and extensively rinsed with distilled water. After photography, the bound staining was eluted with 10% (wt/vol) cetylpyridinium chloride. The alizarin red S in samples was quantified by measuring absorbance at 550 nm and calculated according to a standard curve. 

### 2.7. Western Blot

RAW264.7 cells were used to evaluate the possible signaling pathways [11]. RAW264.7 cells were seeded onto 6-well plates. After reaching confluence, cells were incubated with WL1101 (200 *μ*g/mL) for 30 min and treated with RANKL (10 ng/mL) for different time intervals. Cells were then washed with cold PBS and lysed for 30 min at 4°C with lysis buffer as described previously [11]. For the separation of cytosolic extracts and nuclear extracts, cells were cultured on a 6 cm dish. After reaching confluence, cells were treated with test substances. Cytosolic extracts (CE) and nuclear extracts (NE) were then separated by NE-PER (Nuclear and Cytoplasmic Extraction Reagents, Thermo Scientific-Pierce, Rockford, IL, USA), and the time point was selected according to our previous study [11]. Equal protein (30 *μ*g) was applied per lane, and electrophoresis was performed under denaturing conditions on a 10% SDS gel and transferred to an Immobilon-P (PVDF) membrane (Merck-Millipore, Bedford, MA, USA). The blots were blocked with 5% nonfat milk in TBS-T (0.5% Tween 20 in 20 mM Tris and 137 mM NaCl) for 1 h at room temperature and then probed with antibodies against specific antigens (1 : 1000) at 4°C overnight. After 3 washes by TBS-T, the blots were subsequently incubated with goat anti-rabbit or anti-mouse peroxidase-conjugated secondary antibody (1 : 10000) for 1 h at room temperature. The blots were visualized by enhanced chemiluminescence using Amersham Hyperfilm ECL (GE Healthcare, Upland, CA, USA) or Biospectrum Imaging System (UVP, Upland, CA, USA). For normalization purposes, the same blot was also probed with anti-actin, anti-IKK, or anti-C23 (Nucleolin, NCL) antibody (1 : 1000).

### 2.8. Osteoporosis Induced by Ovariectomy (OVX)

 Three-month-old female Wistar rats (230–270 g) were used in this study. Rats were ovariectomized bilaterally under Zoletil (Virbac, Carros, France)/Rompun (Bayer Animal Health GmbH, Leverkusen, Germany) anesthesia, and control rats were sham operated for comparison. Rats were administered with antibiotic (Baytril, Bayer Animal Health GmbH) after surgery. After three days recovery, ovariectomized rats were randomly divided into three groups. The number of animals is 9, 11, 10, 9 for sham operated, OVX control, OVX treated with 50 mg WL1101, and OVX treated with 200 mg WL110, respectively. All animals were kept under controlled conditions at room temperature (22 ± 1°C) and a 12 h light-dark cycle. Distilled water or WL1101 (50 or 200 mg/kg/day) was administered to rats (once/day) by gastric intubation for 33 days. One-way analysis of variance (ANOVA) and two-tailed Student's *t*-test were used in the statistical analysis of trabecular bone mineral density, trabecular bone morphogenetic indices, and serum markers in animal studies.

### 2.9. Analysis of Bone Resorption and Renal and Liver Markers in Serum

 At the end of experiment, rats were anesthetized and then sacrificed. The blood sample was quickly obtained from left ventricle. Serum samples were prepared by centrifugation. CTX (C-terminal telopeptides of type-I collagen) levels were measured by Serum Rat-Laps ELISA assay for the evaluation of bone resorption (Immunodiagnostic Systems, Boldon Colliery, Tyne & Wear, United Kingdom). Serum GOT (glutamate oxaloacetate transaminase), GPT (glutamic pyruvic transaminase), BUN (blood urea nitrogen), and Cre (creatinine) levels were determined by using an auto dry chemistry analyzer (SPOTCJEM SP-4410, Arkray Inc., Kyoto, Japan).

### 2.10. Microcomputed Tomography (*μ*CT)

 Live rats were scanned on Day 29. On Day 33, rats were sacrificed, and the tibiae and femora were removed and fixed with 4% paraformaldehyde and then analyzed by *μ*CT. Fixed bones were subjected to the X-ray microtomography apparatus by using Skyscan 1176 (SKYSCAN, Kontich, Belgium). Scanning was done at 80 keV and 309 *μ*A with an aluminum plus copper filter. The images were collected at a resolution of 9 *μ*m/pixel for isolated tibiae and femora or 18 *μ*m/pixel for live animals. Reconstruction of sections was carried out with GPU-based scanner software (GPU-NRecon). Quantification of bone mineral density and trabecular morphometric indices was performed in a defined cancellous bone area located 2–4 mm (225 sections) below the growth plate of the proximal end of the tibia and the femur. The analysis was performed by scanner software (CTAn). Trabecular morphology was described by measuring bone volume fraction (bone volume/tissue volume, BV/TV), ratio of bone surface to tissue volume (BS/TV), trabecular number (Tb N), trabecular thickness (Tb Th), and trabecular bone mineral density (BMD). The 3D images were obtained with scanner software (CTvox). 

### 2.11. Statistical Analysis

Each experiment was repeated at least 3 times. All quantitative data were presented as mean ± SEM. Significant differences were determined by ANOVA and 2-tailed Student's *t*-test. A difference was considered significant if *P* < 0.05.

## 3. Results

### 3.1. WL1101 Inhibits Osteoclastogenesis in Primary Cultured Cells

**Figure 1:**
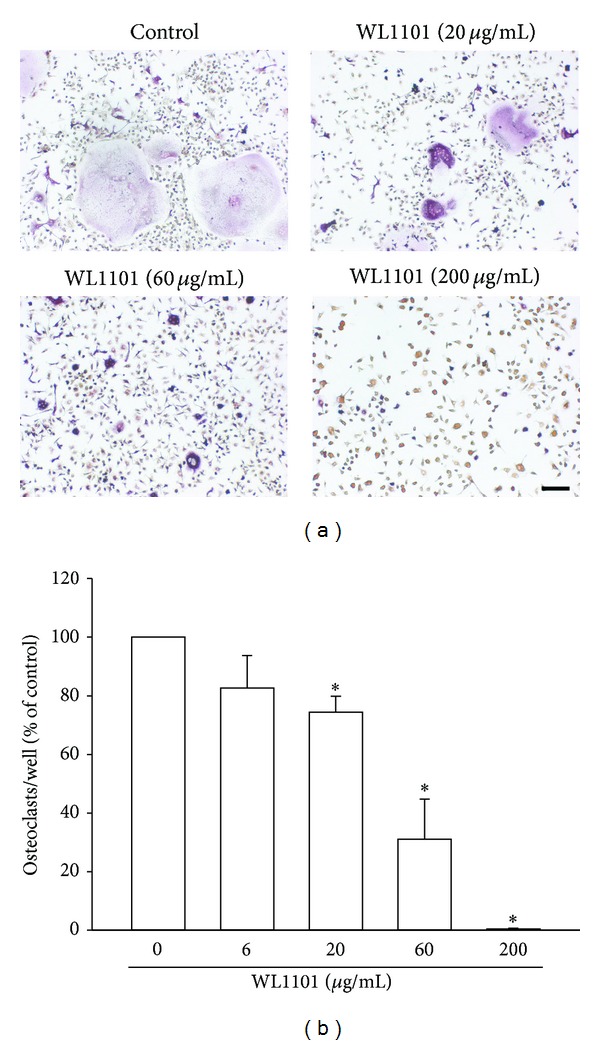
Inhibition of osteoclast differentiation by WL1101 in primary cultured cells. Osteoclast precursors (bone marrow macrophages) isolated from long bone of adult male rats were incubated in the presence of mouse recombinant soluble RANKL (5 ng/mL) and murine M-CSF (20 ng/mL) with or without WL1101 for 5 days. (a) Representative images of TRAP staining showed that treatment with WL1101 exerted a concentration-dependent inhibition on osteoclastogenesis. The quantitative data are shown in (b). TRAP-positive cells containing three or more nuclei were counted. Each value represents mean ± S.E.M. (*n* = 3); *P < 0.05 compared with control vehicle. Scale bar: 100 *μ*m.

**Figure 2:**
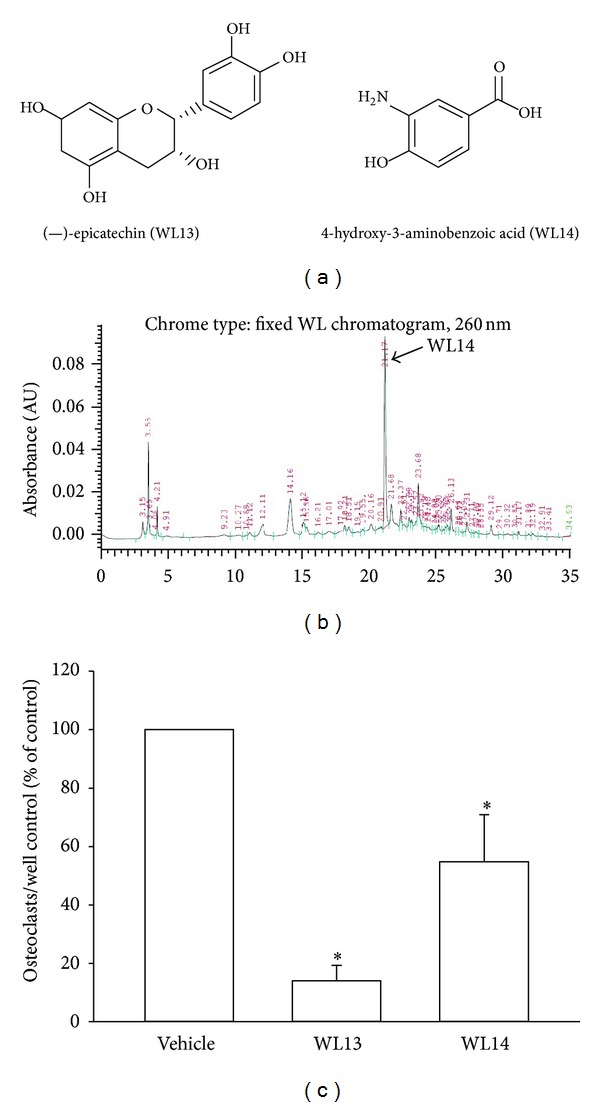
Separation of active compounds from WL1101. Two active compounds were isolated from WL1101. The structures of WL13 and WL14 were shown in (a). (b) HPLC was used to isolate the peak compounds. (c) Osteoclast precursors isolated from long bone of adult male rats were incubated in the presence of mouse recombinant soluble RANKL (5 ng/mL) and murine M-CSF (20 ng/mL) with or without diverse pure compound for 5 days. It was found that WL13 and WL14 can potently inhibit RANKL-induced osteoclastogenesis. Each value represents mean ± S.E.M. (*n* = 3); *P < 0.05 compared with control vehicle.

We first examined the effect of ethanol extracts derived from fresh rhizome of *Davallia formosana*, WL1101, on RANKL-induced osteoclast differentiation. Bone marrow derived hematopoietic cells from long bones of adult male rats were used to study the differentiation of osteoclasts. As shown in Figure 1, WL1101 concentration dependently inhibited TRAP-positive multinucleated osteoclast formation on Day 6 in primary cell cultures. The inhibition was 25.6 ± 5.4%, 69.0 ± 13.7%, and 99.6 ± 0.2% for 20 *μ*g/mL, 60 *μ*g/mL, and 200 *μ*g/mL WL1101, respectively. The IC_50_ is 33.8 *μ*g/mL. We also analyzed the inhibitory effects of isolated pure compounds of WL1101 on RANKL-mediated osteoclastogenesis. HPLC was used to analyze several peak compounds within WL1101 (Figures 2(a) and 2(b)). Here, we found that WL13, (−)-epicatechin or WL14, 4-hydroxy-3-aminobenzoic acid at 10 *μ*M can significantly inhibit osteoclastogenesis (Figure 2(c)). The inhibition was 86.0 ± 5.3% and 45.3 ± 16.1% for WL13 and WL14, respectively.

### 3.2. WL1101 Inhibits RANKL-Induced NF-*κ*B Activation

**Figure 3:**
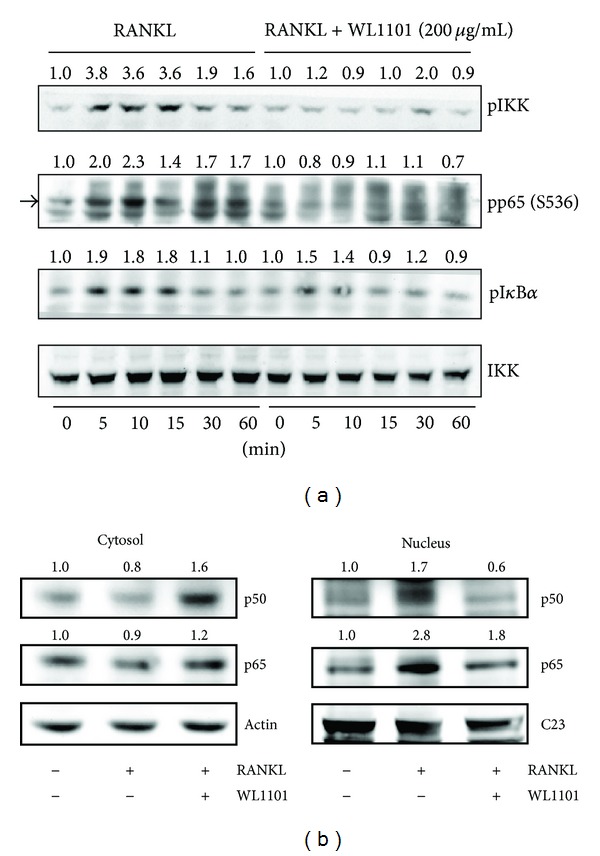
WL1101 inhibits RANKL-induced NF-*κ*B activation in RAW264.7 cells. (a) RAW264.7 cells were preincubated with WL1101 (200 *μ*g/mL) for 30 min and then administered with RANKL (10 ng/mL) at several time intervals. Western blotting showed that treatment with RANKL rapidly increased the phosphorylation of IKK, NF-*κ*B-p65, and I*κ*B*α*. Pretreatment with WL1101 ameliorated the activation of IKK, NF-*κ*B-p65, and I*κ*B*α*. (b) RAW264.7 cells were preincubated with WL1101 (200 *μ*g/mL) for 30 min and then incubated with RANKL (10 ng/mL) for another 30 min. Cytosolic and nuclear extracts were separated by NE-PER. Western blot analysis showed that WL1101 significantly inhibited the translocation of NF-*κ*B subunits of p50 and p65 from cytosol into nucleus. C23 (Nucleolin, NCL) was used for internal control of nuclear protein (*n* = 3).

 It is well known that RANKL plays a crucial role in osteoclast differentiation and bone resorption [12]. We thus further examined the effects of WL1101 on RANKL signaling. We previously reported that application of RANKL rapidly activates NF-*κ*B signaling pathways in RAW264.7 cells [11]. To examine the effect of WL1101 on RANKL-induced NF-*κ*B signaling, the upstream signaling of NF-*κ*B was measured by immunoblotting. As shown in Figure 3(a), treatment with RANKL at 10 ng/mL rapidly increased phosphorylated IKK*α*/*β*, I*κ*B*α* and NF-*κ*B-p65. Pretreatment with WL1101 (200 *μ*g/mL) inhibited RANKL-induced phosphorylation and activation of IKK*α*/*β*, I*κ*B*α*, and NF-*κ*B-p65 (Figure 3(a)). As shown in Figure 3(b), treatment with RANKL for 30 min markedly increased the levels of the NF-*κ*B-p50 and NF-*κ*B-p65 subunits in the nuclear extract. Pretreatment with WL1101 (200 *μ*g/mL) significantly decreased the nuclear translocation of p50 and p65 following RANKL stimulation.

### 3.3. No Significant Effect of WL1101 in Primary Cultured Osteoblasts

**Figure 4:**
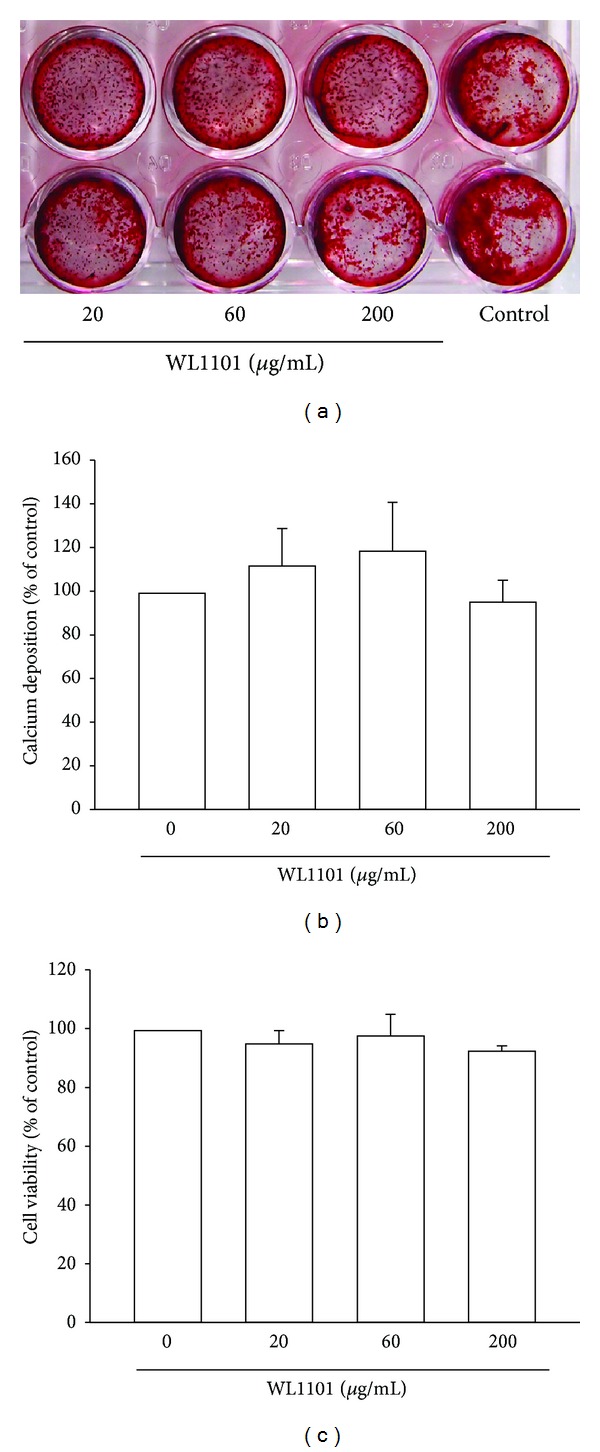
WL1101 exerts no significant effect on calcium deposition and cell viability in primary cultured rat osteoblasts. (a) For calcium deposition analysis, primary osteoblasts were cultured in 24-well plates and exposed to WL1101 in the presence of L-ascorbic acid and *β*-glycerophosphate for 14 days. Long-term treatment with WL1101 exerted no effects on the mineralized deposition of osteoblasts on day 14. The quantitative data are shown in (b) (*n* = 4). (c) For cell viability assay, primary osteoblasts were cultured in 24-well plates and incubated with WL1101 for 2 days. Treatment with WL1101 at 20–200 *μ*g/mL did not affect cell viability (*n* = 3). Each value represents the mean ± SEM.

We further investigated whether administration of WL1101 can regulate the differentiation and cell viability of osteoblasts. Osteoblasts were incubated with osteogenic medium containing L-ascorbic acid and *β*-glycerophosphate in the presence of WL1101 at various concentrations for 14 days. It was found that administration of WL1101 (20–200 *μ*g/mL) exerted little effect on calcium deposition in osteoblasts (Figures 4(a) and 4(b)). In the cell viability assay, the MTT reaction showed that WL1101 exerted little effects on osteoblast viability following 2 days' treatment (Figure 4(c)). These data indicated that WL1101 at these concentrations mainly inhibited RANKL-induced osteoclastogenesis and had less effect on osteoblasts.

### 3.4. Oral Administration of WL1101 Inhibits Ovariectomy-Induced Bone Loss

**Figure 5:**
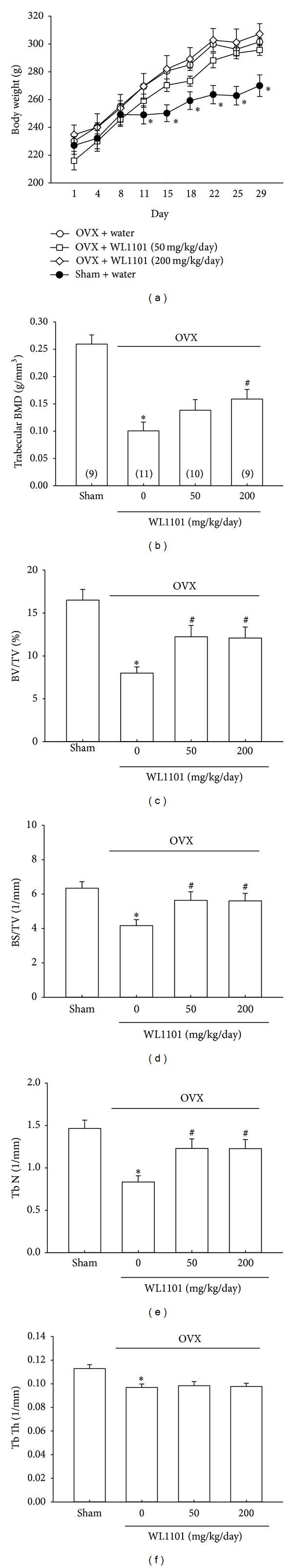
Effects of WL1101 on ovariectomy-induced bone loss in live rats. Distilled water or WL1101 (50 or 200 mg/kg/day) was orally administered to OVX rats via gastric intubation for 33 days (once/day). The body weight was recorded twice a week. On Day 29, the rats were anesthetized by isoflurane, and the right tibia was scanned and analyzed by *μ*CT. (a) The body weight of OVX rats increased compared with sham-operated rats. Administration of WL1101 (50 or 200 mg/kg/day) had no effect on the OVX-induced increase of body weight. (b–f) The right tibia of each live rat was scanned by *μ*CT on Day 29. Administration of WL1101 antagonized the decrease of bone mineral density (BMD) (b), bone volume (c), bone surface (d), and trabecular number (e). Administration of WL1101 exerted less effects on trabecular thickness (f). Each value represents mean ± S.E.M (*n* = 9–11). *P < 0.05 compared with sham-operated group. ^#^ *P* < 0.05 compared with the OVX-control group.

**Figure 6:**
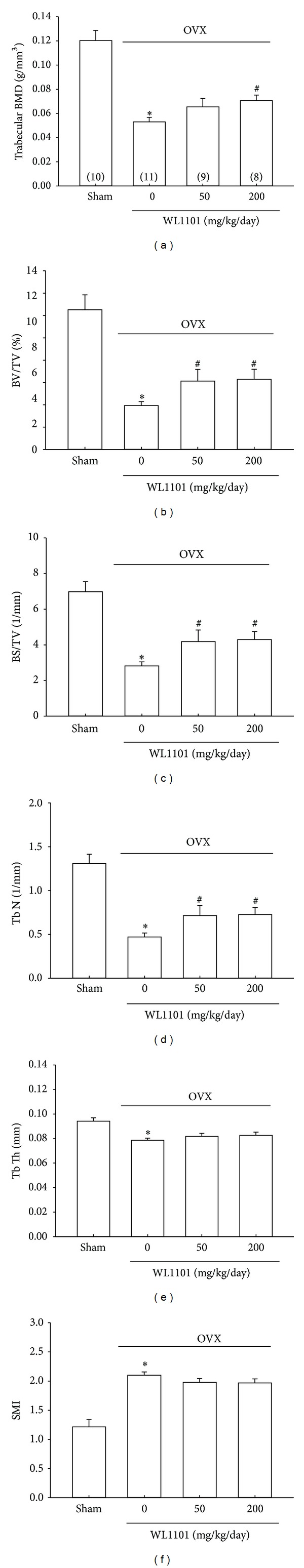
WL1101 inhibits ovariectomy-induced trabecular bone loss in isolated tibia on Day 33. Distilled water or WL1101 (50 or 200 mg/kg/day) was orally administered to OVX rats via gastric intubation for 33 days (once/day). The rats were sacrificed on Day 33, and tibiae and femora were collected. Isolated tibiae were analyzed by *μ*CT. It was shown that administration of WL1101 antagonized OVX-induced decrease of trabecular BMD (a), bone volume (b), bone surface (c), and bone number (d). WL1101 exerted less effects on OVX-induced changes of trabecular bone thickness (e) and structure model index (SMI) (f). Each value represents mean ± S.E.M (*n* = 8–11). *P < 0.05 compared with sham-operated group. ^#^ *P* < 0.05 compared with the OVX-control group.

**Figure 7:**
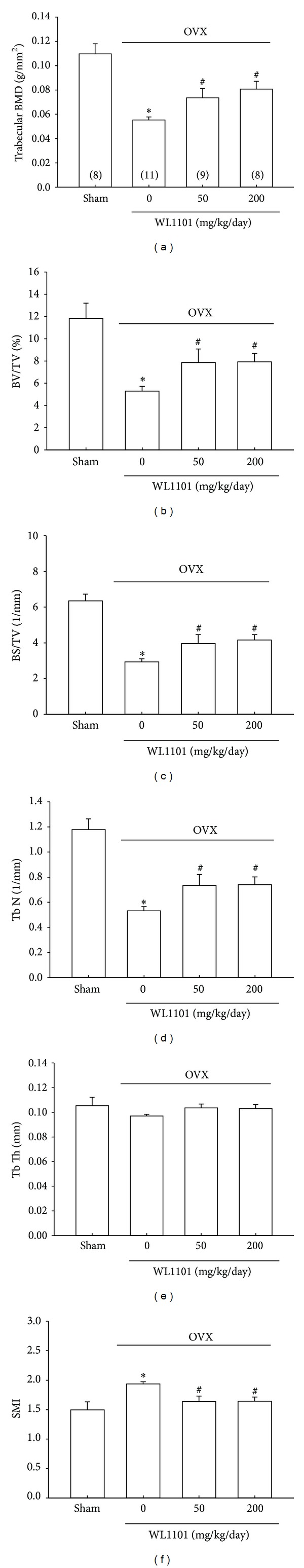
Oral administration of WL1101 ameliorates ovariectomy-induced trabecular bone loss in isolated femur on Day 33. Distilled water or WL1101 (50 or 200 mg/kg/day) was orally administered to OVX rats via gastric intubation for 33 days (once/day). The rats were sacrificed on Day 33, and tibiae and femora were collected for *μ*CT analysis. Administration of WL1101 antagonized OVX-induced decrease of trabecular BMD (a), bone volume (b), bone surface (c), bone number (d), and SMI (f). WL1101 exerted less effects on OVX-induced changes of trabecular bone thickness (e). Each value represents mean ± S.E.M (*n* = 8–11). *P < 0.05 compared with sham-operated group. ^#^ *P* < 0.05 compared with the OVX-control group.

**Figure 8:**
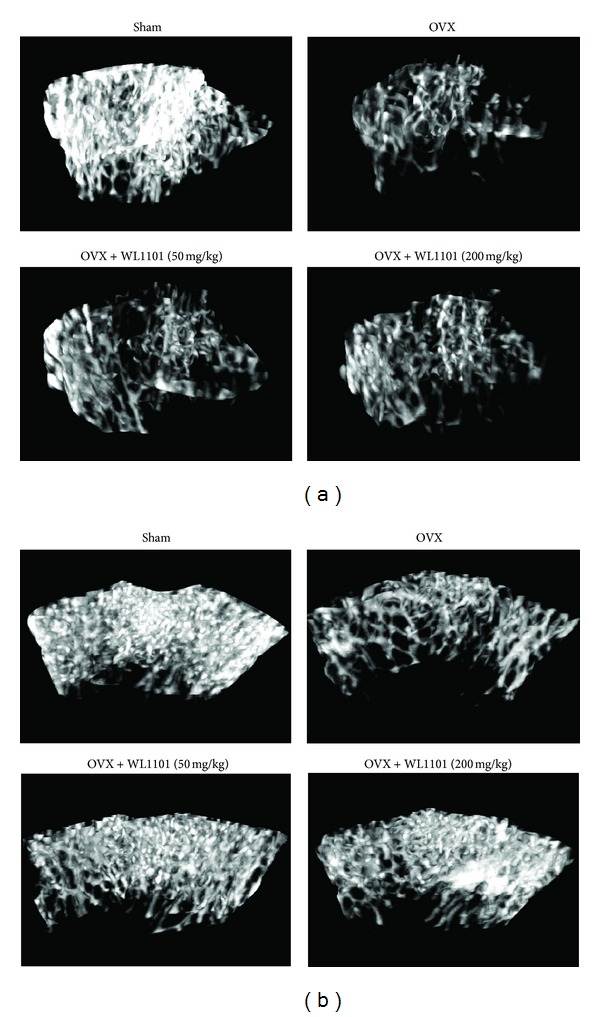
Representative 3D image of spongiosa area in live rat's tibia. Representative 3D images of Figures 6 and 7. It was shown that treatment of WL1101 (50 or 200 mg/kg/day) ameliorated trabecular bone loss in both tibia (a) and femur (b).

**Figure 9:**
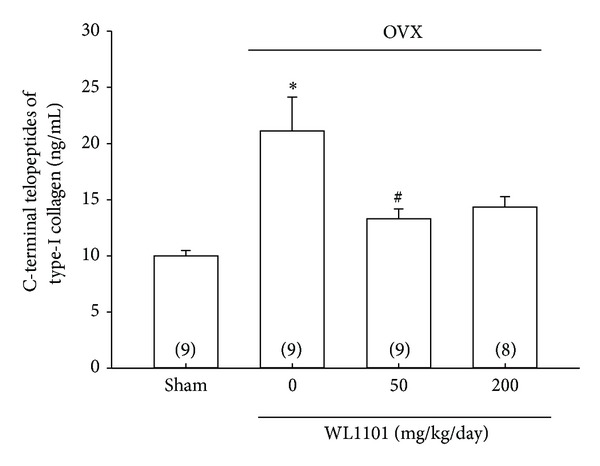
Effects of WL1101 on ovariectomy-induced serum marker of bone resorption in rats. Bone resorption was determined by the serum level of c-terminal telopeptides of type-I collagen (CTX) on Day33 after ovariectomy. WL1101 at 50 mg/kg/day significantly antagonized OVX-induced upregulation of osteoclastic marker. Each value represents mean ± S.E.M (*n* = 8-9). *P < 0.05 compared with sham-operated group. ^#^ *P* < 0.05 compared with the OVX-control group.

 Administration of WL1101 in cell culture showed that WL1101 inhibited RANKL-induced osteoclast differentiation. We further examined the possible effects of WL1101 on osteoclast-mediated bone resorption in animal model. The excess bone resorption was induced in female Wistar rats by ovariectomy (OVX), and WL1101 was then administered daily by gastric intubation (50 or 200 mg/kg/day). During the treatment period, it was found that administration of WL1101 did not significantly influence rats' body weight (Figure 5(a)). The right tibia of live rats was scanned by high resolution microcomputed tomography (*μ*CT, Skyscan 1176) after 4 weeks of treatment of WL1101 or distilled water (on Day-29). As shown in Figure 5, oral administration of two doses of WL1101 (50 and 200 mg/kg/day) ameliorated OVX-induced trabecular bone loss in spongiosa area in tibia (Figures 5(c)–5(e)). Treatment of WL1101 at 200 mg/kg/day also inhibited OVX-induced decrease of trabecular bone BMD (Figure 5(b)). The representative images were shown in Supplementary Figure 1 (see Supplementary Materials available online at http://dx.doi.org/10.1155/2013/647189). At the end point on Day 33, the rats were sacrificed, and tibiae and femora were isolated. The spongiosa area of both proximal tibia (Figure 6) and femur (Figure 7) was analyzed using *μ*CT. Compared to sham-operated rat, trabecular bone mineral density (BMD), bone volume, bone surface, and trabecular number in this area were significantly reduced, and structural model index (SMI) was increased in OVX rats (Figures 6 and 7). Administration of WL1101 at 50 and 200 mg/kg/day significantly antagonized OVX-induced reduction of trabecular bone volume (Figures 6(b) and 7(b)), bone surface (Figures 6(c) and 7(c)), and trabecular number (Figures 6(d) and 7(d)). Administration of 200 mg/kg/day could antagonize OVX-induced decrease of trabecular BMD in both tibia and femur in OVX rats (Figures 6(a) and 7(a)). However, WL1101 did not affect the OVX-induced change of trabecular thickness (Figures 6(e) and 7(e)). In addition, oral administration of WL1101 could inhibit OVX-induced increase of SMI in femur (Figure 7(f)). Representative images are shown in Figure 8. Moreover, to examine the effects of WL1101 on the activity of osteoclasts *in vivo*, the serum concentration of osteoclast marker of CTX (c-terminal telopeptide of type-I collagen) was examined. WL1101 at 50 mg/kg significantly inhibited OVX-induced up-regulation of serum bone resorption marker of CTX after sacrifice of rats on Day 33 (Figure 9). In order to examine the possible toxicity of WL1101 for the homeostasis of blood cells or the function of kidney and liver, whole blood and serum were further examined. Treatment of WL1101 exerted no significant effect on the number of blood cells, platelets, and hemoglobin (Supplementary Figure 2). In addition, administration of WL1101 (50–200 mg/kg/day) did not affect functional markers of kidney (BUN and Cre) and liver (GOT and GPT) (Supplementary Figure 3). 

## 4. Discussion

Excess activity of osteoclasts contributes to many bone diseases including osteoporosis, arthritis, and cancer bone metastasis [13–15]. Osteoporosis is the most common bone disease caused by a relatively increased rate of osteoclast-mediated bone resorption, which exceeds the rate of osteoblast-mediated bone formation and results in a net loss of bone mass. Therefore, antiresorptive agent is a therapeutic strategy of osteoporosis. For example, Prolia (denosumab, GlaxoSmithKline), an efficient monoclonal antibody against RANKL, was recently approved to treat osteoporosis by FDA [16]. 

Here, we examined the possible therapeutic effects of ethanol extracts (WL1101) of *Davallia formosana* on osteoclast-mediated osteoporosis *in vitro* and *in vivo*. In Chinese folk medicine, Drynaria fortunie is called Gu-Sui-Bu, which means that it has potential therapeutic effects on bone fracture. Drynaria fortunie is used for bone diseases including bone fracture, arthritis, and osteoporosis. In Taiwan, both *Davallia formosana* and *Drynaria fortunei* are considered as Gu-Sui-Bu and are used for bone-related disorders. The extracts of rhizome of *Drynaria fortunei* [5–7] and *Davallia formosana* [8] are reported to inhibit the differentiation of osteoclasts and decrease steroid-induced or ovariectomy-induced bone loss in rats. However, the efficacy of the crude extracts of Gu-Sui-Bu in those reports may not be good enough for further therapeutic applications.

In this study, we found that the potency of ethanol extracts derived from fresh rhizome of *Davallia formosana* was much better than extracts from dried rhizome (data not shown). We then chose the fresh rhizome of *Davallia formosana*, which was harvested in Santimen Township (Pingtung County, Taiwan), for further experiment. 

Several kinds of ethanol extracts derived from fresh *Davallia formosana* by diverse methods were tested and we found that WL1101 was the most potent to inhibit RANKL-induced differentiation of osteoclasts. Two active compounds from WL1101 were isolated, WL13 and WL14. WL13, (−)-epicatechin is a similar compound to EGCG [(−)-Epigallocatechin-3-gallate], which is one of the polyphenolic compounds from green tea [17] reported to inhibit the differentiation of osteoclasts. However, the HPLC analysis showed that WL14, 4-hydroxy-3-aminobenzoic acid, which is the richest compound in WL1101, could also significantly inhibit RANKL-induced osteoclastogenesis. Some compounds of modified aminobenzoic acid are considered as inhibitors of osteoclast vacuolar ATPase [18]. Furthermore, our results indicate that WL1101 inhibits RANKL-induced osteoclastogenesis via inhibiting the activation of NF-*κ*B signaling.

In animal studies, we demonstrated that WL1101 at two doses (50 or 200 mg/kg/day) could ameliorate OVX-induced bone loss in rat model. The OVX rat is a widely used model for examining the drug effects on osteoporosis because OVX rats show many similarities in their pathophysiological mechanisms of bone deterioration to human menopausal osteoporosis [19–21].*μ*CT is a powerful tool for analyzing progress of bone loss in animal model [22]. The dose of WL1101 used in rat is 50 mg/kg/day or 200 mg/kg/day, which is equivalent to the dose of 500 mg/60 kg or 2000 mg/60 kg in the adult human [23]. Administration of WL1101 at 50 or 200 mg/kg/day significantly ameliorated OVX-induced decrease in trabecular bone mineral density, bone volume, surface, and numbers in both tibiae and femora which are associated with excess bone resorption. However, previous reports have demonstrated that the efficient dosage of extracts from dried rhizome of Drynaria fortune or from dried rhizome of *Davallia formosana* is about 10 g/kg/day [6] or 500 mg/kg/day [8], respectively. Here, we found that the efficient dosage of WL1101 derived from fresh rhizome of *Davallia formosana* is markedly lower than other extracts of Gu-Sui-Bu as reported previously [6, 8]. The possible toxicity of WL1101 at these doses was also evaluated. Oral administration of WL1101 at these doses did not significantly influence the homeostasis of blood cells and the function of kidney and liver. However, the detailed mechanism regarding how WL1101 can inhibit the differentiation of osteoclast needs further investigation.

In conclusion, our results showed that WL1101, the extracts of fresh rhizome of *Davallia formosana*, can potently and significantly inhibit the differentiation of osteoclasts via interfering with the activation of NF-*κ*B and ameliorate OVX-induced bone loss in rats. Two active compounds were identified and may participate in the actions of WL1101. The ethanol extracts of fresh rhizome of *Davallia formosana* may have therapeutic potential for the treatment of osteoclast-mediated bone diseases.

## Supplementary Material

The supplementary material contain the representative images of trabecular bone of spongiosa area refer to Figure 5. This part also include the analysis of whole blood and serum markers of functions of kidney and liver.
