# Supplementary material for: Ethanol Extracts of Fresh Davallia formosana (WL1101) Inhibit Osteoclast Differentiation by Suppressing RANKL-Induced Nuclear Factor-κB Activation
Source: Evid Based Complement Alternat Med. 2013 Sep 28;2013:647189. doi: 10.1155/2013/647189 (PMC3804452; doi:10.1155/2013/647189)
Supplement: Supplementary file 1 — The supplementary material contain the representative images of trabecular bone of spongiosa area refer to Figure 5. This part also include the analysis of whole blood and serum markers of functions of kidney and liver. [file 647189.f1.docx]

**Supplementary figure legend:**

**S Figure 1.** **Representative 3D image of spongiosa area in live rat’s tibia**

Representative 3D images of figure 5. It was shown that treatment of WL1101 (50 or 200 mg/kg/day) ameliorated trabecular bone loss in tibia. **(a)** Sagittal view **(b)** Transaxial view.

**S Figure 2. No effect of WL110 on blood cell numbers in whole blood analysis**

Distilled water or WL1101 (50 or 200 mg/kg/day) was orally administered to OVX rats via gastric intubation for 33 days (once/day). The whole blood was collected and analysed on Day-33. It was found that treatment of WL1101 exert no effect on the number of white blood cells **(a)**, red blood cells **(b)**, platelets **(c)** and the level of haemoglobin **(d)**. Each value represents mean ± S.E.M (n=3-6).

**S Figure 3. No effect of WL110 on serum markers of renal or liver function**

Distilled water or WL1101 (50 or 200 mg/kg/day) was orally administered to OVX rats via gastric intubation for 33 days (once/day). The serum of rats was collected and analysed on Day-33. It was shown that oral administration of WL1101 did not affect blood urea nitrogen **(a)**, creatinine **(b)**, which are markers of renal function and levels of GOT **(c)** and GPT **(d)**, which are markers of liver function. Each value represents mean ± S.E.M (n=8-12).

**Supplementary figures:**


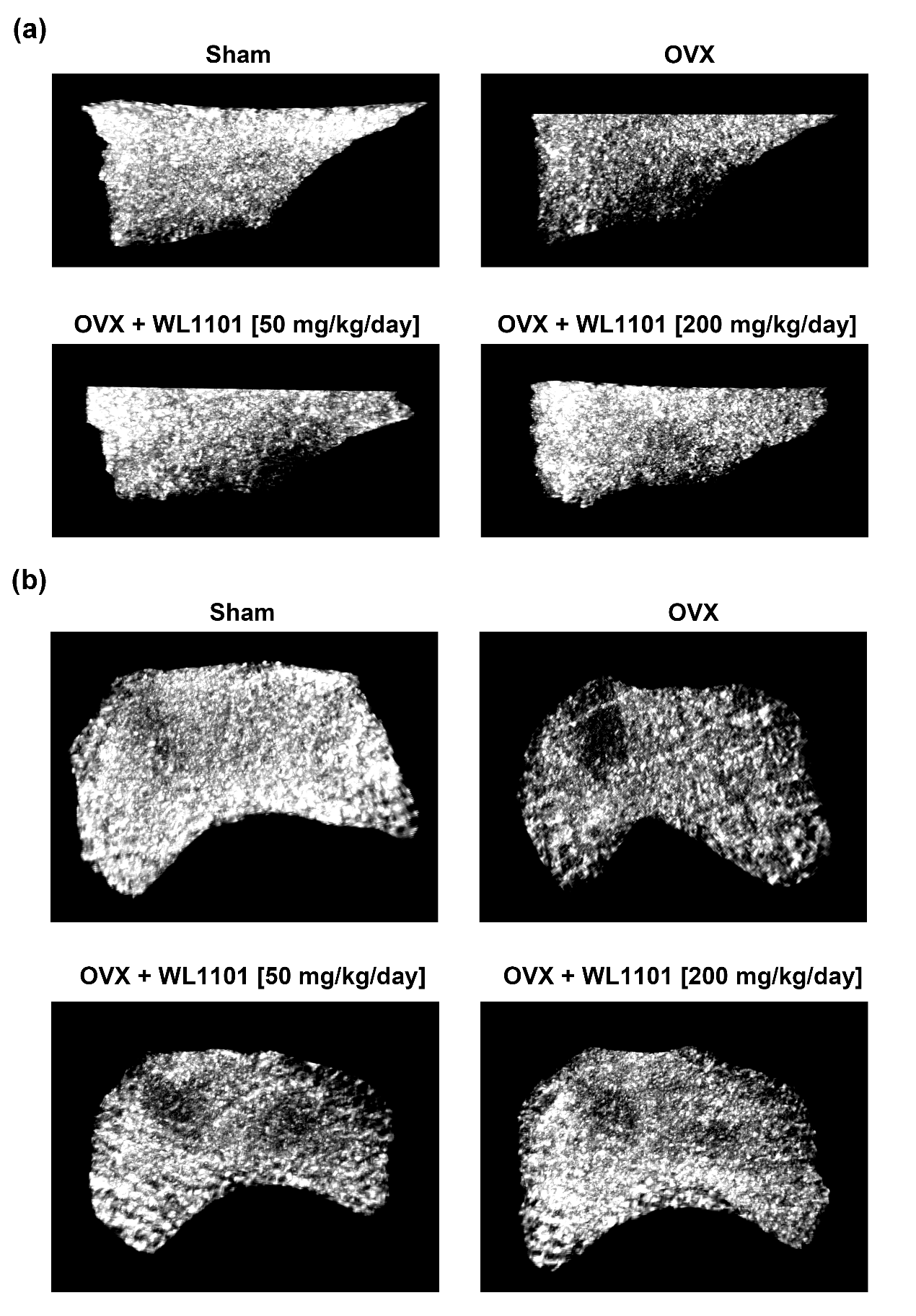


S Figure 1.


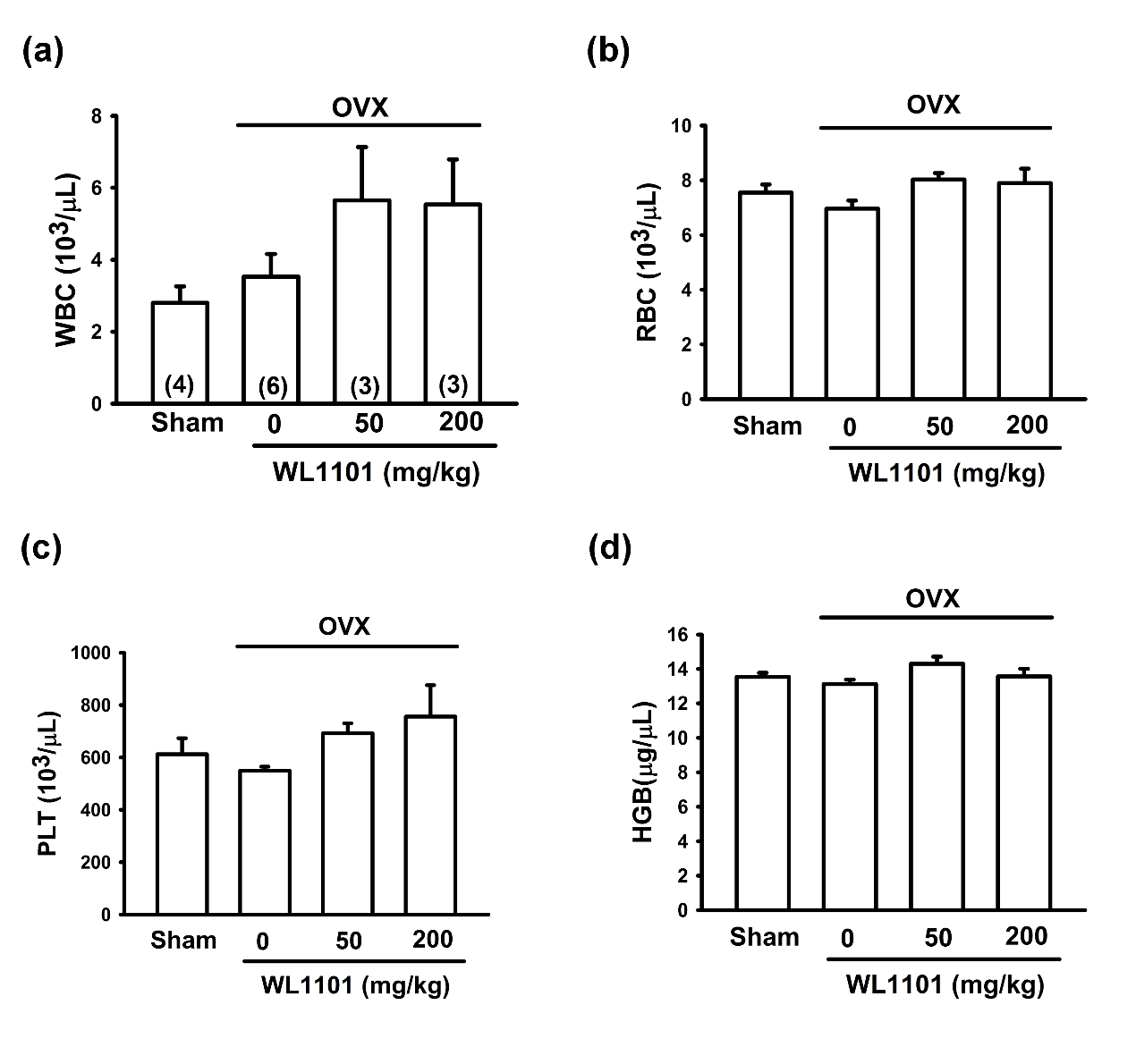


S Figure 2.





S Figure 3.
